# Reconstitution of Pol II (G) responsive form of the human Mediator complex

**DOI:** 10.3906/biy-2009-12

**Published:** 2021-06-23

**Authors:** Murat Alper CEVHER

**Affiliations:** 1 Department of Molecular Biology and Genetics, Faculty of Science, Bilkent University, Ankara Turkey; 2 Visiting Assistant Professor, Laboratory of Biochemistry and Molecular Biology, The Rockefeller University, New York USA

**Keywords:** Mediator, Pol II (G), Gdown1, transcription, reconstitution, multibac

## Abstract

RNA polymerase II (Pol II) is a 12 subunit protein complex from yeast to human that is required for gene expression. Gdown1 containing Pol II [Pol II (G)] is a special form of Pol II that is catalytically inactive and heavily depends on the 30-subunit Mediator complex for its activator and basal dependent function in vitro. Here we report for the first time, the identification and the generation of a 15-subunit human Mediator complex via the novel multibac baculovirus expression system that is fully responsive to Pol II (G). Our results show complete recovery of Pol II (G) dependent transcription both with full 30-subunit Mediator and also with 15-subunit recombinant Mediator that we synthesized. Moreover, we also show that the recombinant Mediator interacts with Pol II (G) as well. These results enlighten us towards understanding how a certain population of Pol II that is involved in selected gene regulation is activated by Mediator complex.

## 1. Introduction

Mediator complex is a multisubunit coactivator complex consisting of different modules and is the chief regulator of the transcription by RNA polymerase II (Pol II) (Allen and Taatjes, 2015; Robinson et al., 2016). It was first discovered as a purified complex in yeasts (Kim et al., 1994). Studies have shown that Mediator complex has an essential protein composition and structure which is largely conserved from yeast to human (Myers and Kornberg, 2000; Malik and Roeder, 2003). Human Mediator complex was initially annotated as thyroid hormone receptor-associated protein (TRAP) complex (Malik and Roeder, 2003). Subsequently, it was found that the Mediator complex has a critical role in not only activator dependent transcription but also basal transcription. Furthermore, Mediator complex-depleted nuclear extracts have been shown to be defective in terms of RNA Pol II transcription function in both TATA promoters and TATA-less promoters (Mittler et al., 2001; Lacombe et al., n.d.).

Human Mediator is a dynamic coactivator complex consisting of 30 subunits with a molecular size of 2MDa (Tsai et al., 2014). It consists of 4 modules: head, middle, tail and kinase (Tsai et al., 2014). In humans, the head and middle modules, together with MED14 and MED26, constitute the active core Mediator complex (Cevher et al., 2014). The tail and kinase modules are connected dynamically to the core Mediator (Blazek et al., 2005). The Mediator complex primarily transmits signals from general transcription factors and activators to Pol II, regulating the function of the enzyme in the preinitiation, initiation, elongation and reinitiation stages (Yin and Wang, 2014; Allen and Taatjes, 2015; Jeronimo and Robert, 2017).

The eukaryotic Pol II, consisting of 12 evolutionally conserved subunits, is responsible for transcription of all protein-encoding genes (Malik and Roeder, 2010). It has been found that Pol II also has a 13th subunit called Gdown1 (encoded by POL2RM), and the Pol II form containing this subunit is called Pol II (G) (Hu et al., 2006). Gdown1 persists to bind tightly to Pol II, even in the presence of high concentrations of salts or urea at low molarity. This protein is metazoan specific and is expressed in a wide number of human tissue samples (Fagerberg et al., 2014). Therefore, Gdown1 is thought to play important roles in transcriptional regulation. Also, in vitro studies have shown that the Gdown1 acts as a repressor by inhibiting the transcription activity of Pol II in the absence of the Mediator complex (Hu et al., 2006). 

In the presence of the Mediator, however, the inhibitory effects of Gdown1 on both activator-dependent and -independent (basal) transcription disappear (Hu et al., 2006; Jishage et al., 2012). Unfortunately, the biological role of Gdown1-mediated repression and how the Mediator reverses this repression are not fully understood yet. However, it was found that in the presence of Gdown1, TFIIF’s (an important factor on PIC assembly) binding to Pol II was inhibited, leading to a blockage of PIC assembly (Jishage et al., 2012). Also, Gdown1 directly binds to RPB1 and RPB5 subunits of Pol II and competes with TFIIF. In another interaction study, it has been suggested that the region (C-terminal) where Gdown1 interacts with Pol II coincides with TFIIF and TFIIB binding sites on Pol II (Jishage et al., 2018). Binding of Gdown1 to Pol II inhibits transcription by preventing TFIIF and TFIIB interaction with Pol II in the absence of Mediator (Jishage et al., 2018). Gdown1 also plays roles in the elongation phase of transcription. Since Gdown1 and TFIIF interaction sites on Pol II overlap, Gdown1 prevents the stimulation of elongation by TFIIF and blocks termination activity of TTF2. Phosphorylation of Gdown1 (Ser-270) by kinase decreases the binding of Pol II and reverses the inhibition of TTF2 and TFIIF (Cheng et al., 2012; Guo et al., 2014). As this form of Pol II is responsive to Mediator and requires it for function, it is critical to understand which subunits of the Mediator are required to overcome this Gdown1 negative affect on Pol II. 

In this study, in vitro transcription assays using RNA polymerase II enzyme in the form of Pol II (G) purified from nuclear extracts and Mediator complexes with varying compositions (head, middle, head + middle, head + middle + 14) produced with multibac system were performed. Our results have shown for the first time that both the recombinant Mediator with 15 subunits and the full natural Mediator complex containing 30 subunits exerted a full recovery of transcription in the presence of Pol II (G). Moreover, we also show that this function of the core Mediator comes from its ability to interact with Pol II (G) that finally results in recruiting Pol II to promoters (Cevher et al., 2014).

## 2. Materials and methods

### 2.1. Purification of Pol II (G)

HEK293 nuclear extracts were prepared from FLAG-tagged Gdown1 stable cell line and dialyzed in different concentration of TGEA buffer (with salt concentrations of 100, 150, 200 and 300 mM) and fractionated on a DE52 column. The fractionated nuclear extract (TGEA 300) dialysate was then subjected to M2 agarose beads and eluted with 0.5 mg/mL FLAG peptide.

The input, flow-through, and the eluates obtained with various concentrations of the elution buffer in the purification steps were analyzed with Western blot, in which antibodies to Gdown1, RPB1 and RPB6 were used. Polyacrylamide gel electrophoresis was performed to check the purity after FLAG peptide elution and the gel was analyzed by silver staining.

#### 2.1.1. Purification of activators, coactivators and general transcription factors

General transcription factors were purified as described before (Cevher et al., 2014). Basically, while the recombinant TFIIB, TFIIE and TFIIE were expressed in bacteria and purified, baculovirus expresse TFIIA as well as the activators Gal4VP16, TRa and RXRa were purified from insect cells and purified (Cevher et al., 2014). Full natural Mediator, TFIID, TFIIH and Pol II were purified from HeLa cell lines stably expressing FLAG-tagged subunit of the corresponding protein complexes (Cevher et al., 2014).

### 2.2. Reconstitution and purification of the human Mediator complex

The Head+Middle+14+26 complex was reconstituted as described by Cevher et al. (2014). The cDNAs encoding different Mediator subunits which were tagged with either FLAG, Myc, 6xHis or HA tags were cloned into pFBDM transfer vectors. Head module subunits contained pFBDM (MED6, MED8, MED11, f-MED17, MED18, MED20, MED22, MED30) and the middle module subunits contained pUCDM (MED4, HA-MED7, MED9, His-MED10, Myc-MED21, MED31). The plasmids were then integrated into the bacmid DNA and the viruses were amplificated by transfecting Sf9 cells. After that, Hi5 cells were infected for the production of H+M and H+M+14 complex. 

Protein extraction was performed using BC500 [500 mM KCl, 20 mM Tris-Cl, pH 7.9, 20% glycerol, 0.1 mM EDTA (pH 8.0), 0.1 mM PMSF and DTT] from Hi5 cells 60 h after infection and the lysates were washed with BC300 [300 mM KCl, 20 mM Tris-Cl, pH 7.9, 20% glycerol, 0.1 mM EDTA (pH 8.0), 0.1 mM PMSF and DTT]. Subsequently, the extract was incubated with anti-FLAG M2 agarose beads and the proteins were purified by eluting with 0.5 mg/mL FLAG peptide and BC300. Coomassie brilliant blue stain was used to confirm the presence of proteins in the final elution.

### 2.3 In vitro transcription assay

Purified factor- or nuclear extract-based systems were used in the in vitro transcription assays as described previously (Cevher et al., 2014). For the transcription reaction, 50 ng template was used. All templates containing the G-less cassettes downstream of the adenovirus major late (ML) core promoter were used for transcription analysis. The pG5ML template contained five Gal4 binding sites; the p4TRE template contained four T3 response element regions; p4ERED53 template contained four estrogen response element regions and TRE3.

Purified general transcription factors (TFIIA, TFIIB, TFIID, TFIIE, TFIIF, TFIIH) and PC4 are used for the assay. Gal4-Vp16, T3/TR/RxR and TR/RxR were added to the reaction mixtures containing the pG5ML, p4TRE and TRE3 templates, respectively, and labelled nucleotide triphosphates either [α-32P]UTP or [α-32P]CTP. The mixtures were incubated at 30 ºC for 50 min. Electrophoresis was then performed and analyzed by autoradiography.

## 3. Results

### 3.1. Reconstitution of Pol II (G) responsive form of the Mediator complex

**Figure 1:**
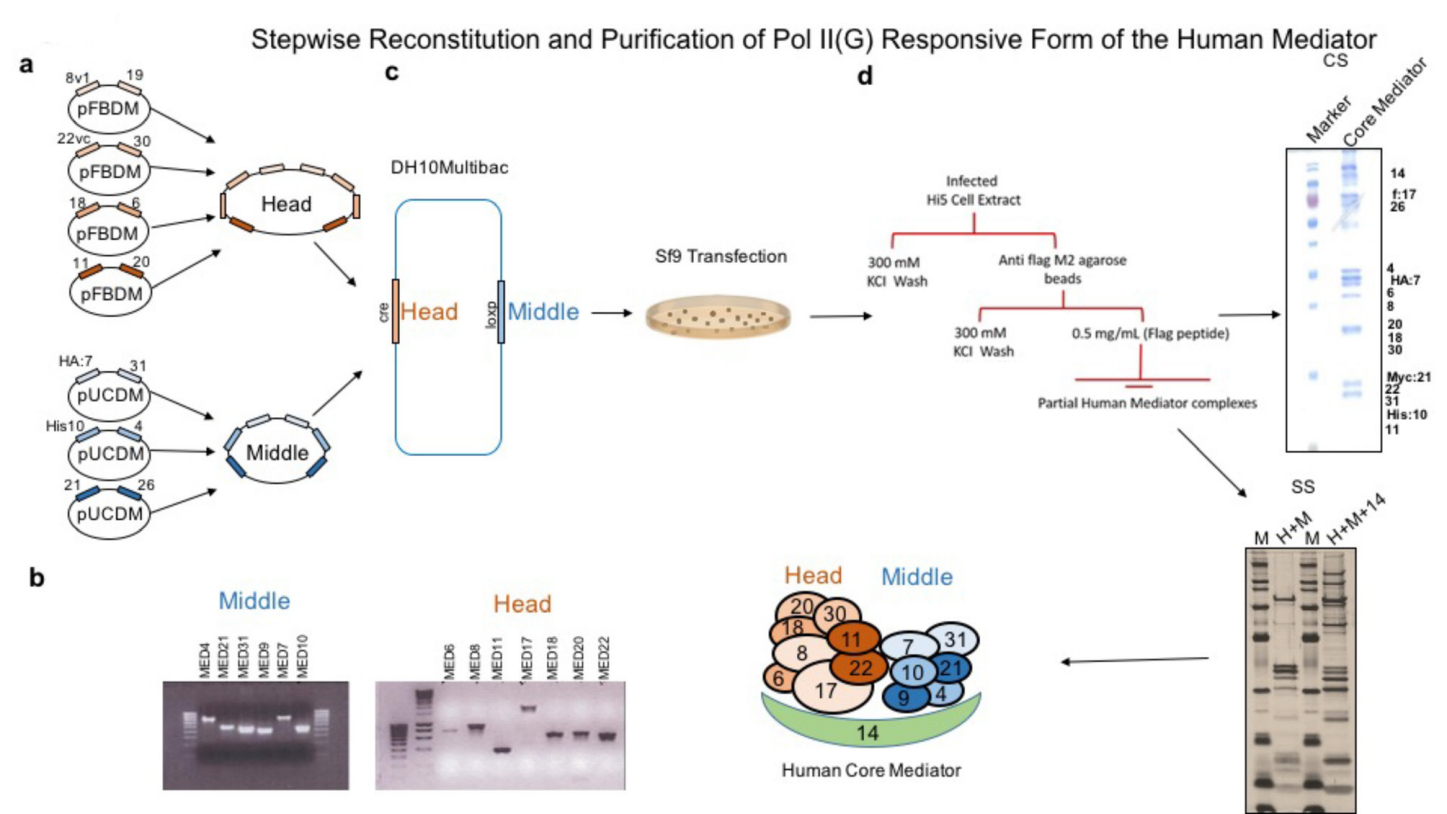
Reconstitution of Pol II (G) responsive form of the human Mediator. (a) Recombinant human head module subunits (MED17, MED8, MED20, MED30, MED11, MED22, MED6 and MED18) and human middle module subunits (MED7, MED21, MED31, MED10, MED4, MED26 and MED9) and the linker MED14 were cloned into pFBDM and pUCDM transfer vectors respectively. (b) PCR product of human Mediator head and middle module subunits cloned in to pFBDM and pUCDM transfer plasmids. (c) The transfer plasmids bearing the head and middle module subunits were transformed into DH10 Multibac containing bacteria. Bacmid was isolated from DH10 Multibac bacteria and transfected to Sf9 insect cells. (d) Protein lysates were prepared and incubated with M2 agarose beads. M2 agarose bound proteins were washed and eluted with FLAG peptide and ran on Superose 6 gel filtration column for further purification. Finally, purified proteins were visualized by coomassie (CS) or silver stain (SS).

RNA Pol II consists of twelve evolutionarily conserved subunits and is required for transcription of protein coding genes. Some fraction of purified Pol II contains a tightly associated and metazoan specific thirteenth subunit called Gdown1 protein. This protein inhibits transcription of Pol II in the absence of the Mediator complex. Here we show the stepwise purification of recombinant human Mediator complex that removes the negative effect of Gdown1. For this, we use the novel multibac baculovirus expression system and recombinantly generate the largest human protein complex that has ever been reconstituted. We basically cloned the human head module subunits (MED17, MED18, MED22, MED11, MED30, MED20, MED6 and MED8) and human middle module subunits (MED31, MED21, MED10, MED31, MED7, MED4, MED9) along with MED14 into pFBDM and pUCDM transfer vectors (Figure 1a). Later, we verified the cloned subunits within these plasmids by PCR (Figure 1b). Upon verification of the cloned subunits, we transformed them in to DH10Multibac containing bacteria. We later purified the DH10 Multibac bacmid from bacteria, verified that the transfer vectors integrated in to the bacmid via transposition as well as Cre-lox P recombination and then transfected insect Sf9 cells with the purified bacmid (Figure 1c). Finally, we extracted proteins from insect cells and purified our recombinant Mediator proteins via affinity purification (against FLAG tag) followed by Superose 6 gel filtration (size exclusion chromatography) to get pure and near homogenous proteins (Figure 1d). 

### 3.2. Reconstitution of Pol II (G) form of the polymerase from HEK293 cell extracts

**Figure 2:**
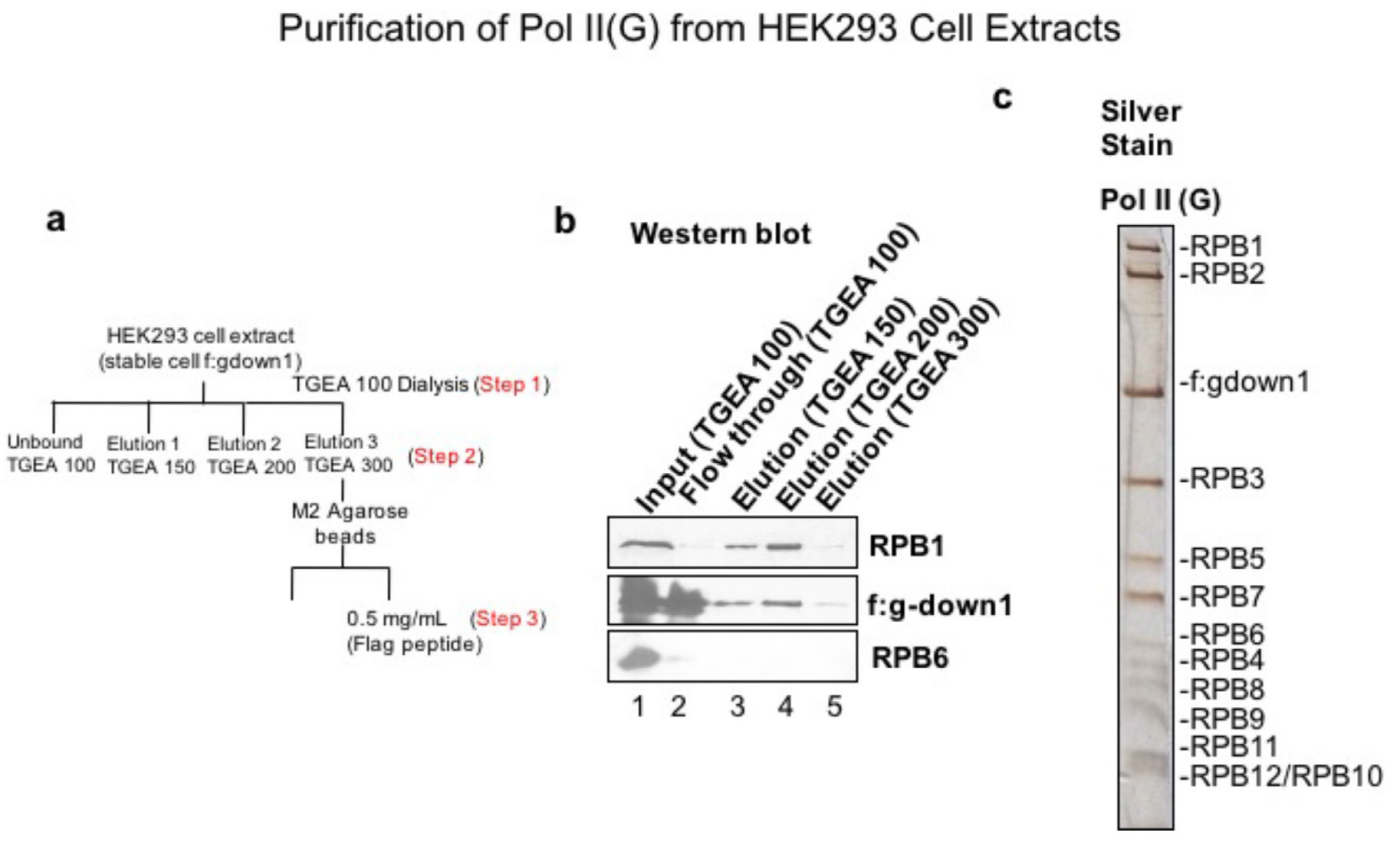
Purification of Pol II (G) from HEK293 cells stably expressing f:Gdown1. (a) Cell extracts were made from HEK293 Cells stably expressing FLAG-tagged Gdown1 protein. The extract was initially dialyzed to 100 mM TGEA and loaded to DE52 ion exchange resin. Bound proteins were eluted with 100 mM, 150 mM, 200 mM and 300 mM TGEA buffer. (b) Western blot showing each elution step of Pol II (G) containing cell extract with TGEA. (C) The 300 mM TGEA fraction was collected and incubated with M2 agarose beads. Later M2 agarose bound proteins were eluted with FLAG peptide and resolved in SDS page gel.

Hek293 cell extracts were stably expressed with f:Gdown1 protein in order to saturate Pol II with Gdown1 and purify large quantity of Pol II with saturated Gdown1. As the tag is on Gdown1, purification of Pol II via FLAG tag ensures that 100% of purified Pol II contains Gdown1 protein. The cell extract was prepared and dialyzed against TGEA with 100 mM salt. Later, the sample was loaded to DE52 anion exchange column (Figures 2a and 2b). The bound proteins were eluted and the fraction with 300 mM TGEA was collected and put to M2 agarose for FLAG tag affinity purification. The purified protein was then resolved in SDS page and silver stain showing all subunits of Pol II along with Gdown1 was observed (Figure 2c).

### 3.3. In vitro transcription with recombinant Mediator

**Figure 3:**
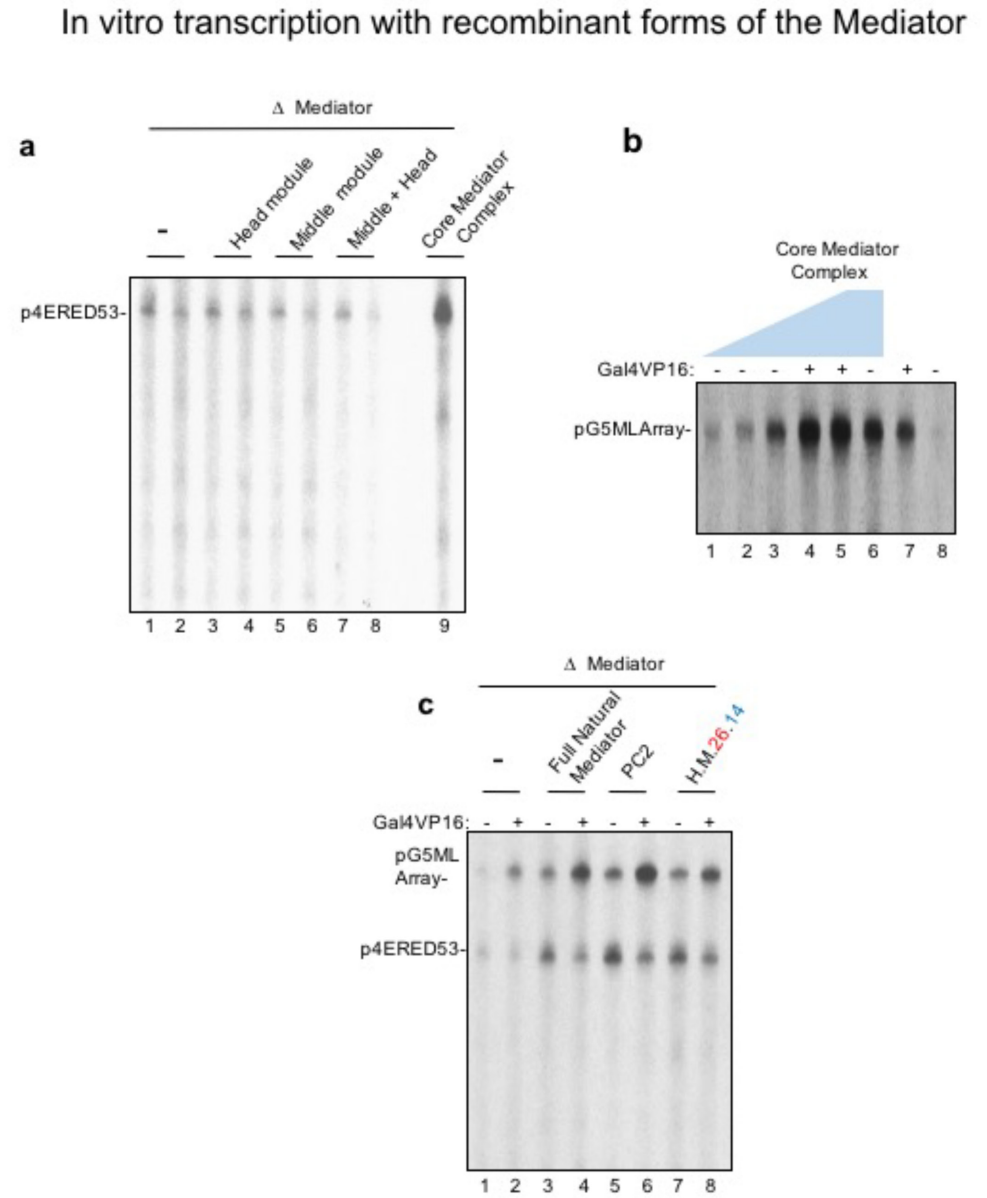
Pol II (G) responsive recombinant human Mediator was tested for transcription with HeLa nuclear extracts bearing Pol II (G). (a) HeLa nuclear extracts were depleted from Mediator with beads crosslinked with MED30 antibody. This antibody can remove the entire 30-subunit Mediator from nuclear extracts. Recombinant Mediator modules (head, middle, head-middle and core [head-middle and MED14)] were supplemented to Mediator depleted HeLa nuclear extracts in order to observe recovery of transcription. (b) HeLa nuclear extracts depleted with Mediator was supplemented with increasing concentration of recombinant human core-Mediator complex from (a) that fully responded to transcription. (c) In vitro transcription was done as in (a) except this time activator Gal4VP16 was supplemented to in vitro transcription reactions.

Prior to checking the activity of our recombinant Mediator with Pol II (G), we characterized its function with HeLa nuclear extracts where the source of transcription comes from HeLa extracts. Basically, we immunodepleted Mediator from HeLa nuclear extracts and performed in vitro transcription reactions by supplementing the Mediator depleted nuclear extracts with our recombinant Mediator complexes. As the nuclear extract has a population of Pol II and Pol II (G), we did in vitro transcription with nuclear extracts and characterized if our recombinant Mediators could recover transcription. As can be seen in Figure 3a, neither head, nor middle nor head+middle could recover transcription (lanes 1–8). However, our human core Mediator complex could recover transcription to its full potential (lane 9). Later, we checked if the transcription was dose dependent on the recombinant human core Mediator complex and saw that as we increased the concentration of this protein complex, we observed more and more transcripts (Figure 3b). Finally, we checked if our recombinant Mediator could recover activated transcription with VP16. For this, we used the natural 30-subunit Mediator complex as a positive control as it fully responds to VP16. As VP16 does not interact with the core-Mediator complex, we expected not to see an activated recovery of transcription but basal transcription (Figure 3c, compare lanes 3–4 to lanes 7–8). This way we verified that our recombinant Mediator is fully functional.

### 3.4. In vitro transcription with activators TR/RXR nuclear extracts and purified system with Pol II (G)

**Figure 4:**
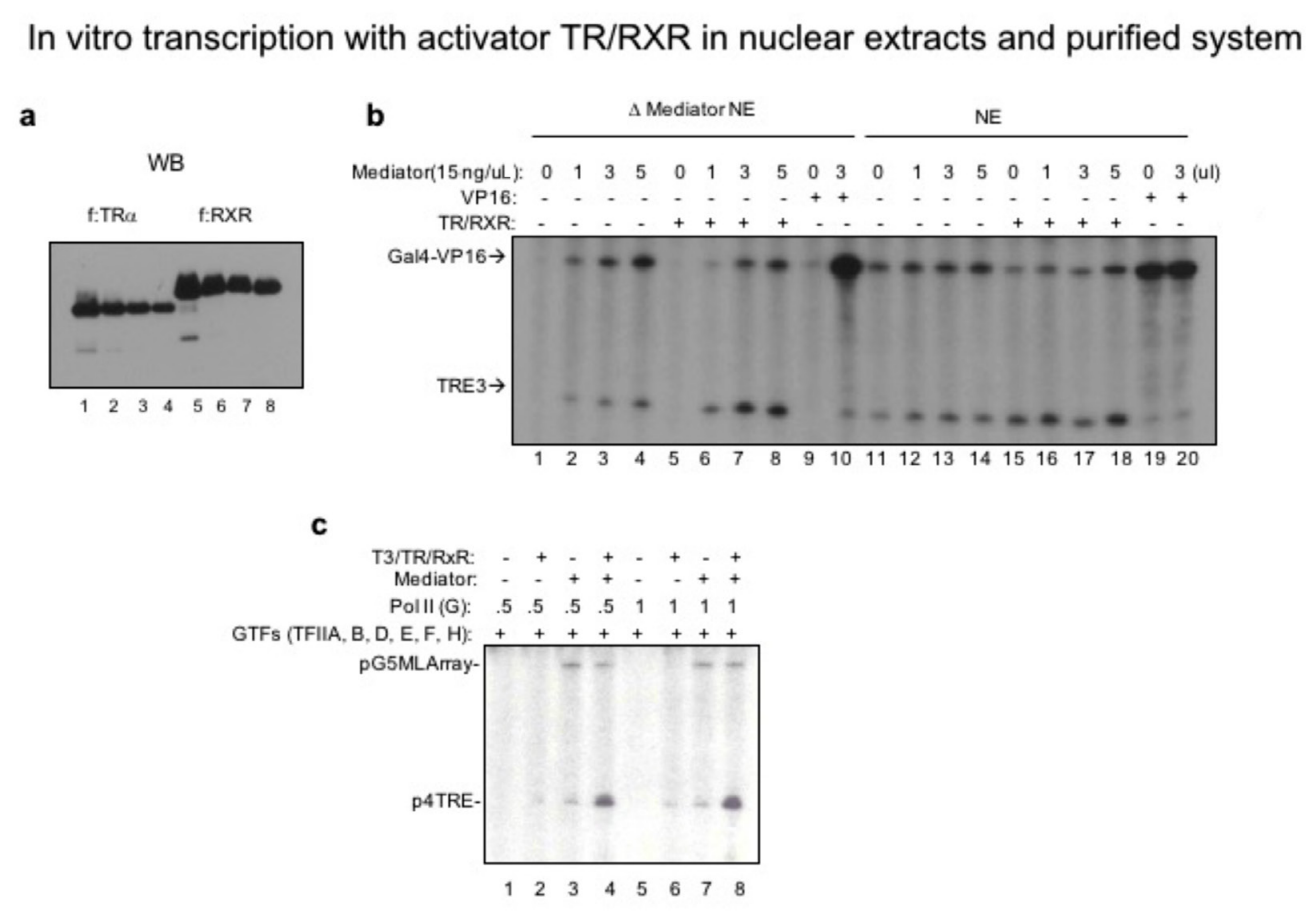
Purification of TR/RXR, in vitro transcription with TR/RXR and TR/RXR-Pol II (G). (a) Western blot of purified FLAGtagged thyroid hormone receptor and retinoid X receptor. (b) In order to validate function for TR/RXR and the purified Mediator complex and to set-up a functional transcription assay, in vitro transcription assay was conducted on Gal4-VP16 responsive pG5HML and TR responsive 3TRE templates. Lanes 1–10 were performed with Mediator depleted extracts while lanes 11–20 were conducted with mock (control) treated NE. Supplementation of purified Mediator and activators fully recovered and enhanced transcription. (c) In vitro transcription was conducted as in (b) this time with purified general transcription factors (GTF) and purified Pol II (G) to validate their activity. Basically, purified GTFs (TFIIA, TFIIB, TFIID, TFIIE, TFIIF and TFIIH), purified Pol II (G) and Mediator was used along with the activators TR and RXR to test the activity of Pol II (G).

Human Mediator was initially purified from liganded thyroid hormone receptor (TR/RXR). As Mediator strongly interacts with this activator and fully responds in transcription, we recombinantly generated and purified thyroid hormone receptor and its partner retinoid X receptor (Figure 4a). We next tested if this liganded activator responds to Mediator dependent transcription in the presence (Figure 4b, lanes 1–10) and absence of Mediator (Figure 4b, lanes 11–20). For that we used either Mediator depleted or not depleted nuclear extracts. As can be seen, the activator TR/RXR (lanes 3 vs. 7) and VP16 (lanes 9 vs. 10) fully responded to Mediator dependent transcription. Once we optimized the working conditions for Mediator and the activator in nuclear extracts that partially has Pol II (G), we next purified all the general transcription factors (TFIIA, TFIIB, TFIID, TFIIE, TFIIF and TFIIH), PC4, Mediator and Pol II (G) and optimized transcription with varying amounts of Pol II (G) in the presence and absence of Mediator and TR/RXR (Figure 4c). With the Mediator, the negative effect of Gdown1 was fully recovered for both basal (lanes 1 vs. 3 and 5 vs. 7) and activated transcription (lanes 2 vs. 4 and 6 vs. 8). 

### 3.5. Recombinant human core Mediator complex removes Gdown1 negative effect on Pol II and associates with Pol II (G)

**Figure 5:**
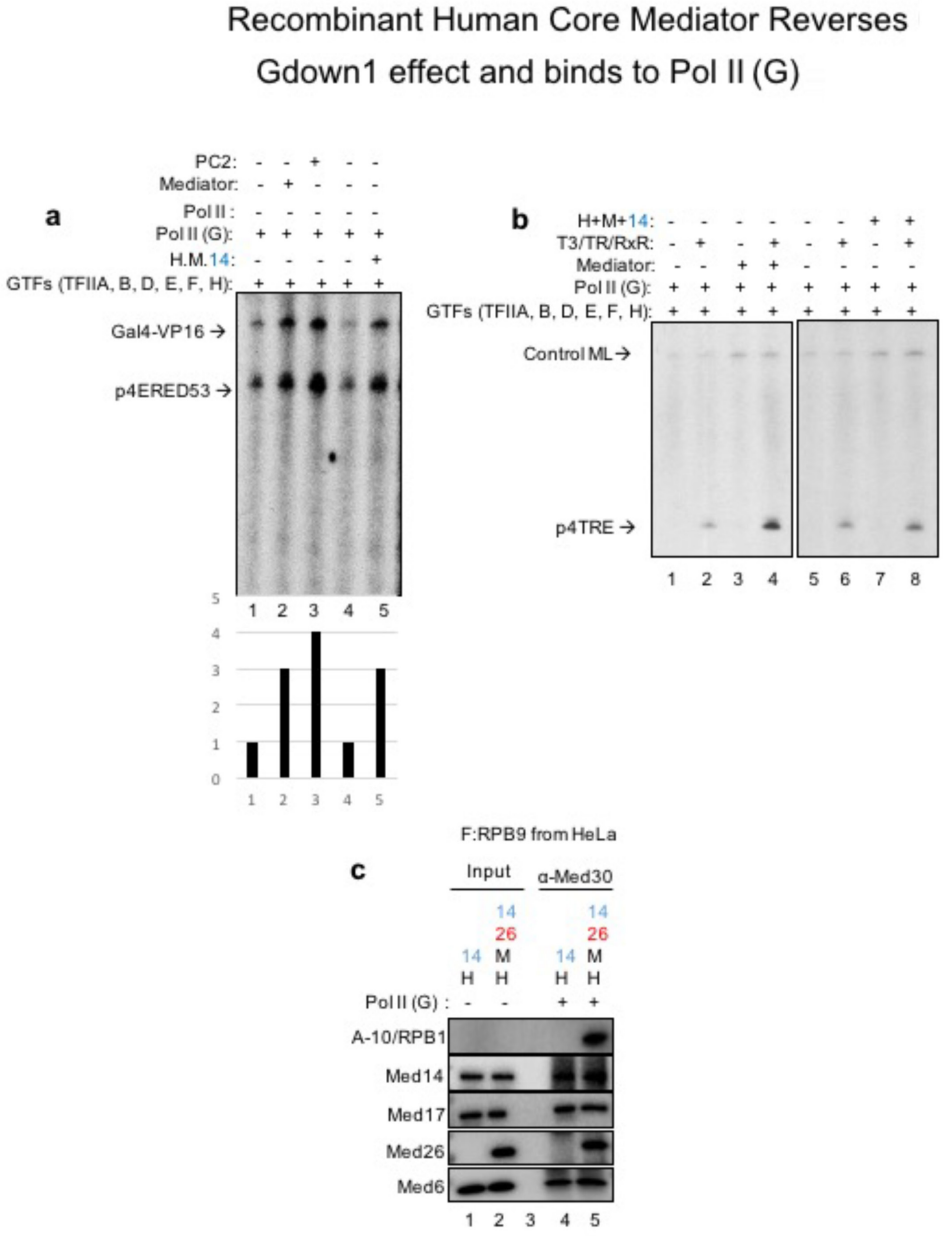
Functional and mechanistic characterization of Pol II (G) responsive form of recombinant Mediator. (a) Purified GTF’s were used here along with recombinant core and endogenous purified full length Mediator complex to test the recovery of Gdown1 negative effect on Pol II. Natural Mediator (lanes 1–2), PC2 Mediator (kinase module lacking form of endogenous Mediator, lane 2) and recombinant core Mediator complex (lanes 4–5) was tested for recovery of basal level transcription done with Pol II (G) (lanes 4–5). Gdown1 free Pol II was used as a control to show activity of recombinant Mediator and the negative effect of Gdown1 (lanes 6–7). (b) In vitro transcription was done as in (a). This time TR/RXR was used to observe activated transcription in the presence of either natural Mediator (lanes 1–4) and recombinant core Mediator (lanes 5–8). (c) Core Mediator association with Pol II (G) was tested via immunoprecipitation. Head+14 served as a negative control.

Finally, we wanted to characterize if our recombinant human core Mediator complex could revert the negative effect of Gdown1 on Pol II. In order to do these kinds of experiments, our lab is equipped with unique recombinant purified system where we can dissect in to purified proteins (general transcription factors) to understand the necessity/function of each factor in the in vitro transcription system. Therefore, we purified all GTFs (Cevher et al., 2014), PC4 and recombinant Mediator variants among with Pol II (G) (Figure 2) and tested if the recombinant Mediator could revert the negative effect of Gdown1 on Pol II just like a full natural 30-subunit Mediator can. Remarkably, for the first time, we can see from Figure 5a that the recombinant human core Mediator could recover basal transcription of Pol II (G) to the same extent as full natural 30-subunit Mediator. This suggests us that the subunits within this 16 subunit core Mediator could revert the negative effect of Gdown1 and may not need the tail and the kinase modules to overcome this effect. Next, we checked if the recombinant human core Mediator could recover TR/RXR activated transcription. Here, as we know that our human core Mediator does not interact with TR/RXR, we do not expect it to recover activated transcription but we expect it to only recover basal transcription. As can be seen from Figure 5b, while the natural Mediator could recover both activated and basal transcription (lanes 1–4), the recombinant core Mediator could only recover basal transcription (lanes 5–8) proving once again that our human core Mediator fully responds to Pol II (G) and removes the negative effect. Mediator exerts its function mainly by binding to RNA Pol II and recruiting it to promoters. Finally, in order to understand the mechanism of how the recombinant human core Mediator activates Pol II (G) transcript, we checked if this recombinant Mediator could interact with Pol II (G). Remarkably, for the first time, we see that this form of the Mediator interacts to its full extent with Pol II (G) (Figure 5c, lanes 4 vs. 5) and telling us this is how it can recruit this form of Pol II to promoters (Figure 6).

## 4. Discussion

Mediator complex is critical for both basal and activated transcription as it serves as a hub between enhancer bound activators and promoter bound general transcription factors. In fact, Mediator was first isolated in yeast with Pol II and thus was called a holoenzyme. Its strong association with Pol II is a key property of Mediator as it brings Pol II to promoters to facilitate transcription (Cevher et al., 2014). Later, Mediator was purified from human cell extracts through liganded thyroid hormone receptor and hence was initially named as thyroid hormone receptor-associated protein complex (TRAP). Later, we recombinantly generated the core-Mediator complex composing of head+middle+MED14 to bind to Pol II and recruit Pol II to promoters to facilitate transcription. A long standing question was in what is the Mediator component that could revert the negative effect of Gdown1 protein in Pol II (G). Previously, we reconstituted the largest protein complex up to date and showed that the recombinant human Mediator can fully activate Pol II mediated transcription (Cevher et al., 2014). Here, in order to see if our recombinant core could recover transcription of Pol II (G) and compare it to the full natural Mediator complex, we tested our recombinant human core-Mediator complex for in vitro transcription and coimmunoprecipitation assays. Remarkably, our in vitro transcription assays as well as coimmunoprecipitation show that the recombinant Mediator not only can bind to Pol II (G) but also can revert its negative effect on transcription just like the natural Mediator can. Our findings now open-up new avenues towards understanding the Mediators role at its subunit, modular and multimodular level in detailed understanding of not only how enhancer bound activated genes are activated (Cevher et al., 2014) but also how the negative effects are overcome by the Mediator complex. 
